# Dysgerminoma with a Somatic Exon 17 *KIT* Mutation and SHH Pathway Activation in a Girl with Turner Syndrome

**DOI:** 10.3390/diagnostics10121067

**Published:** 2020-12-10

**Authors:** Ada Gawrychowska, Ewa Iżycka-Świeszewska, Beata S. Lipska-Ziętkiewicz, Dominika Kuleszo, Joanna Bautembach-Minkowska, Marcin Łosin, Joanna Stefanowicz

**Affiliations:** 1 Department of Paediatrics, Haematology and Oncology, Clinical University Centre, 7 Debinki Street, 80-952 Gdansk, Poland; agawrychowska@uck.gda.pl; 2 Department of Pathology and Neuropathology, Faculty of Health Sciences, Medical University of Gdansk, 3a Maria Sklodowska-Curie Street, 80-210 Gdansk, Poland; ewa.izycka-swieszewska@gumed.edu.pl; 3 Centre for Rare Diseases, Medical University of Gdansk, 7 Debinki Street, 80-952 Gdansk, Poland; b.lipska@gumed.edu.pl; 4 Clinical Genetics Unit, Department of Biology and Medical Genetics, Faculty of Medicine, Medical University of Gdansk, 1 Debinki Street, 80-211 Gdansk, Poland; 5 Department of Biology and Medical Genetics, Faculty of Medicine, Medical University of Gdansk, 1 Debinki Street, 80-211 Gdansk, Poland; dominika.kuleszo@gumed.edu.pl; 6 Department of Paediatrics, Diabetology and Endocrinology, Clinical University Centre, 7 Debinki Street, 80-952 Gdansk, Poland; bauti@gumed.edu.pl; 7 Department of Surgery and Urology for Children and Adolescents, Faculty of Medicine, Medical University of Gdansk, 1-6 Nowe Ogrody Street, 80-803 Gdansk, Poland; m.losin@gumed.edu.pl; 8 Department of Paediatrics, Haematology and Oncology, Faculty of Medicine, Medical University of Gdansk, 7 Debinki Street, 80-210 Gdansk, Poland; 9 Faculty of Health Sciences, Medical University of Gdansk, 3a Maria Sklodowska-Curie Street, 80-210 Gdansk, Poland

**Keywords:** Turner syndrome, somatic exon 17 KIT mutation, growth hormone treatment, dysgerminoma, HH signaling

## Abstract

This article reports a case of a 7-year-old girl with Turner syndrome, treated with growth hormone (GH), who developed ovarian dysgerminoma. The patient karyotype was mosaic for chromosome Xq deletion: 46,X,del(X)(q22)/45,X. No Y chromosome sequences were present. Molecular studies revealed the presence of a driving mutation in exon 17 of the KIT gene in the neoplastic tissue, as well as Sonic-hedgehog (SHH) pathway activation at the protein level. The patient responded well to chemotherapy and remained in complete remission. This is the first case of dysgerminoma in a Turner syndrome patient with such oncogenic pathway.

## 1. Introduction

Turner syndrome (TS; [1]) is a chromosomal disorder resulting from complete or partial X-chromosome monosomy. More than half of such cases are mosaic cases featuring normal and abnormal cell lines and/or abnormal chromosomes including isochromosome i(Xq), ring chromosome (r(X)), partial losses in one X chromosome, or Y chromosome fragments. The clinical features are heterogeneous, and dysmorphic features are often mild or absent. Severe growth impairment is the hallmark of the disease, and growth hormone (GH) therapy is recommended to be introduced early [2]. Women with Turner syndrome seem to be at increased risk for different types of malignancy, which may stem from genetic and hormonal factors [3]. The early loss of ovarian function (ovarian hypofunction or premature ovarian failure) is very common in TS because the majority of patients have gonadal dysgenesis [4]. Certain TS patients who have a mosaic karyotype containing Y chromosome material are at increased risk for gonadal neoplasia, the most frequent of which is gonadoblastoma, a precursor lesion that can progress into malignant germ cell tumors (GCTs), mainly of the dysgerminoma type [4,5,6,7]. Therefore, a bilateral gonadectomy is recommended in all TS patients carrying a Y-chromosome [2].

The pathogenicity of ovarian GCT is largely heterogenous with significant age-dependency. Several theories of GCT have been proposed, such as the arrest of gonocyte migration and maturation, blockade of apoptosis, mitosis–meiosis switch alterations, miRNA dysregulation, neoplastic niches, and signaling pathway changes. Several pathways are involved in gonadogenesis and intragonadal germ cell–stroma interactions. One of them is crucial in embryogenesis—Hedgehog (Hh) signaling, whose misactivation occurs in different human tumors. Moreover, few cases of mutations in Hh coding genes have been reported to play a role in gonadal dysgenesis and are associated with GCT formation [8,9,10,11].

In prepubertal girls, the most common GCTs include mature teratoma and yolk sac tumors, while in postpubertal females, typical ovarian lesions are teratomas and dysgerminomas. Most cases of dysgerminomas occur in the second to third decade of life, but 10% of cases develop in children with dysgenetic gonads. Few available studies show that somatic chromosome 12p abnormalities, common in testicular GCT, are also present in the majority of ovarian dysgerminomas. Another somatic genetic abnormality is the pathogenic variants in *KIT*. Two distinct but overlapping pathways of ovarian GCT development exist. In the first pathway, the triggering factor is the presence of constitutional Y chromosome material in individuals with defects in their sexual development (including male-to-female sex-reversal cases in patients with 46,XY complete gonadal dysgenesis) who present clinically as phenotypic females. The second results from spontaneous KIT activation leading to increased survival and the proliferation of undifferentiated oogonia [9,12,13].

## 2. Case Presentation

A 7-year-old patient was born full term with a birth weight of 2600 g and a body length of 54 cm. At the age of 4 years, the patient’s height was 95.2 cm (<3rd percentile), her weight was 11.6 kg (<3rd percentile), and her body mass index (BMI) was 12.12 (below −2 SDs). Mild dysmorphic features, including a high-arched palate and a low hairline at the nape of the neck, were observed. Hypothyroidism requiring L-thyroxin supplementation was diagnosed. Further evaluation revealed the mos 46,X,del(X)(q22)[11]/45,X[19] karyotype leading to a clinical diagnosis of TS. GH treatment with 5 mg somatropin (Omnitrope Pen Cartridge Sandoz GmbH, Kundl, Austria) once daily was initiated 4 months after the diagnosis of TS. The GH dose corresponded to the levels of insulin-like growth factor 1 (IGF-1) and insulin-like growth factor binding protein 3 (IGFBP3) in the patient’s serum. During GH-therapy, the IGF-1 and IGFBP3 levels were always in the normal range for the patient’s age and gender. A good response and accelerated growth were observed.

Two years and 8 months after the initiation of GH therapy, the patient was admitted to a hospital due to fever and abdominal pain lasting for three days. Physical examination revealed a palpable mass in the lower abdomen. Upon ultrasonography, the presence of a heterogeneous tumor of 6 × 4 × 9 cm with calcifications was confirmed. Laboratory tests showed increased levels of lactate dehydrogenase (LDH) (568 U/L), Ca125 (39.7 U/mL), fibrinogen (5.61 G/L), D-dimer (5198 µg/L FEU), and C-reactive protein (CRP) (13.87 mg/L). The serum beta-hCG, alpha-fetoprotein (AFP), Ca15.3, and Ca19.9 were within normal limits. The contrast MRI demonstrated a well-defined 65 × 69 × 95 mm solid and cystic mass likely originating from the ovary and infiltrating the peritoneum, accompanied by enlarged retroperitoneal and iliac lymph nodes. The uterus was relatively small, and evident ovaries were not identified bilaterally. GH therapy was then stopped.

**Figure 1:**
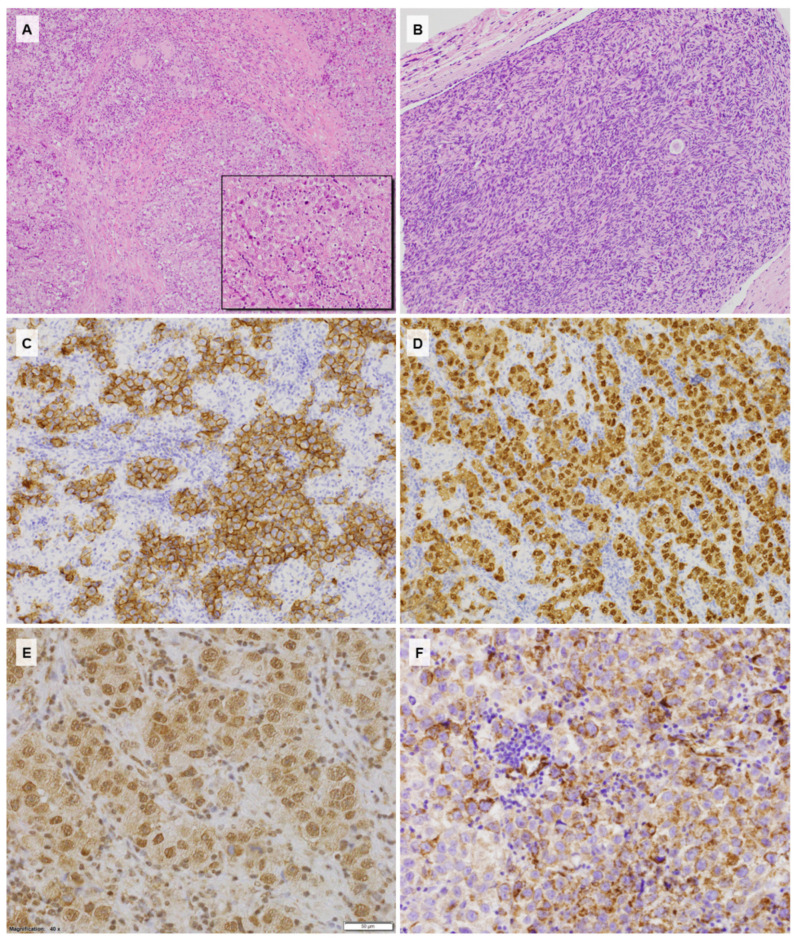
(**A**) Histology of dysgerminoma with stromal fibrous bands and small reactive granulomas (HE, 100×); (**B**) histology of the streak gonad contralateral to the tumor showing the ovarian stroma with a single primordial follicle (HE, 200×); (**C**) strong CD117 expression with membranous enhancement within the neoplastic cells (CD117, 200×); (**D**) Oct3/4 immunoreactivity in the dysgerminoma cells (Oct3/4, 200×); (**E**) Gli1 expression showing strong nuclear and low cytoplasmic reactions (Gli1, 400×); (**F**) SHH cytoplasmic immunostaining within the neoplastic component and negative stromal lymphocytes (SHH, 400×).

A laparoscopic biopsy of the neoplastic mass was then performed. Microscopically, the tumor consisted of nests and sheets of uniform large oval cells with prominent nucleoli and low mitotic activity, surrounded by lymphocytic infiltrate. The histology and immunophenotype (positivity for Oct3/4, CD117, and placental alkaline phosphatase (PLAP), as well as negativity for CD30, Glypican 3, and cytokeratins) were consistent with a diagnosis of dysgerminoma (Figure 1).

Subsequently, the patient received three cycles of chemotherapy with vinblastine, bleomycin, and cisplatin according to the French protocol TGM 95—Strategie therapeutique des tumeurs germinales malignes extra-cerebrales de l’enfant. A partial radiological response to the treatment was observed. An ultrasound exam after the third chemotherapy cycle showed a partial reduction in the tumor. The patient underwent a bilateral salpingo-oophorectomy and an iliac lymph node resection. Histopathological examination revealed complete regressive changes within the tumor and reactive lymph nodes without metastases. The opposite gonads with diameters 13 × 9 × 6 mm contained fibrous tissue and ovarian stroma with very few primary follicles corresponding to a streak gonad.

DNA was extracted from the paraffin embedded tumor sample and subject to a mutational analysis of the 25 genes associated with GCT pathogenesis and/or encoding key proteins of the Hh pathway (Table 1) using the next generation sequencing technique (NGS) with a custom-designed QIAseq-targeted DNA panel (Qiagen, Hilden, Germany). High-throughput sequencing was performed using the NextSeq platform (Illumina, San Diego, CA, USA). The assays were performed following the manufacturer’s instructions. Data analysis, including variant filtering, was performed in line with the recommendations of the Association for Molecular Pathology, American Society of Clinical Oncology and College of American Pathologists [14]. A known pathogenic missense variant in *KIT* NM_000222.2: c.2466T>A (COSM1321; rs121913514) resulting in the amino-acid substitution of asparagine with lysine in the codon 822 at the *hot-spot* exon 17 of the gene was present with a 15% variant allele frequency. In parallel, analysis of the 23 STR *loci* ruled out the presence of chromosome Y (PowerPlexY23; Promega, Madison, WI, USA) in DNA extracted from the tumor sample.

In addition, SHH pathway protein expression was examined with a panel of antibodies by Abcam (GLI1, GLI2, GLI3, PTCH1, SMO, SUFU, SHH) using the routine immunohistochemistry technology DAKO Agilent. The neoplastic cells showed SHH cytoplasmic staining, nuclear GLI1, PTCH1, SUFU expression, as well as a membranous SMO reaction in parallel with GLI2 negativity.

### The Ethical Statement

The study was conducted in accordance with the Declaration of Helsinki, and the protocol was approved by the Ethics Committee of the Medical University of Gdansk, Poland (NKBBN/597/2013, MUG). The parents of a child gave their informed consent for inclusion before they participated in the study.

## 3. Discussion

The etiology of TS involves the dysregulation in meiosis signaling to germ cells, which may result in nondisjunction and monosomy X due to separation failure of the chromosomes in either the parental gamete or during early embryonic divisions. Growth failure affected almost all TS patients, and their final height was approximately 20 cm shorter than the mean adult height. The initiation of GH therapy in TS is recommended early, at approximately 4–6 years of age [2]. GH treatment is associated with concerns about safety, including the risk of neoplasia. Reports suggest no increased risk of primary tumor development in patients without risk factors who are treated with GH, although the issue remains controversial [15,16]. An SAGhE European cohort study comprising 23,984 patients treated with recombinant GH showed no significant carcinogenic effects of r-hGH but indicated an increased risk of bone and bladder cancer in the intermediate risk group including TS patients [17]. Cianfarani assessed the risk of cancer in patients treated with r-hGH based on the available literature and found this matter controversial, as the reports contained conflicting data [18]. One review reported a slightly higher prevalence of de novo malignancies in patients with TS treated via GH than in patients treated with GH for a different reason [19]. Beyond gonadoblastoma and GCTs, TS patients seemed to be at increased risk of various malignancies. A national cohort study of 3425 women diagnosed with TS showed a higher prevalence of central nervous system tumors (especially meningiomas), childhood brain tumors, and cancers of the bladder and urethra compared to the general population. Between 15 and 44 years of age, the incidence of endometrial cancer was also increased, with a significant decrease in breast cancer. The overall risk of cancer was similar to that in the general population [3]. Another cohort study reported that women with TS had an increased risk of melanoma and central nervous system tumors, mainly meningiomas [20].

Approximately 10% of TS patients are mosaic for Y-chromosome material in their genomes [21]. Detecting Y-chromosome sequences is extremely important because the presence of such sequences is associated with an increased risk of gonadoblastoma and GCTs. This risk is estimated at 12% in TS patients with Y-chromosome material and at 1% in patients without Y-chromosome material [6]. Standard karyotyping may not reveal Y-chromosome fragments; thus, more sensitive techniques, such as PCR or fluorescence in situ hybridization (FISH), are often used. The most recent guidelines for the care of women with TS recommend a PCR evaluation only if the patient presents with masculinization and is negative for the Y chromosome in standard karyotyping or FISH [2]. Nevertheless, even if there is no detectable Y-chromosome material in the peripheral blood lymphocytes, this material may be present in other tissues due to skewed mosaicism. Therefore, other sources of DNA (cultured fibroblasts, buccal swabs, or hair follicles) are sometimes tested to search for cryptic Y-chromosome material if the patient presents with masculine features [2]. The percentage of mosaicism often differs between tissues, as patients with low-level mosaicism of the Y-chromosome cell line in peripheral blood lymphocytes may have an increased percentage of this cell line in the gonads, which correlates more directly with the risk of GCTs [22]. However, the prevalence of Y-chromosome material in the gonads may also differ in the gonadal regions, so it cannot be precisely evaluated prior to gonadal removal [22,23]. In the present patient, the presence of the Y-chromosome in the tumor was ruled out using a forensic technique aimed at the identification of even a low fraction of Y material in the tested samples. Therefore, the typical Y-associated pathway of GCT development in TS was not applicable here.

Conversely, comprehensive genetic studies of the neoplastic tissue revealed the presence of a somatic pathogenic variant in exon 17 of the *KIT* gene (NM_000222.2: c.2466T>A (COSM1321; rs121913514)) encoding the growth factor receptor important for normal germ cell migration and development. As the result of a somatic mutation leading to the activation of KIT, sustained survival, proliferation, adhesion, and motility were observed. The detected pathogenic variant resulted in a gain-of-function alteration of the c-KIT receptor. A recent study of 87 ovarian GCTs revealed a homogeneous profile of this group of tumors, with recurrent somatic exon 17 KIT mutations in 16.7% of all samples including 44.4% of dysgerminomas and mixed forms with a dysgerminoma component [24]. Activating *KIT* mutations are considered the primary recognized driving mutation behind ovarian GCT development [12,24,25]. In the studied tumor, no other somatic event was identified, which is in line with the observation that ovarian GCTs have a low somatic mutation rate. Overall, the rate in GCT was ~100× lower than that observed in common adult cancers. This is consistent with an embryonal origin and might also explain the chemosensitivity even in advanced or recurrent disease, as such GCTs are less likely to harbor clones with drug resistance.

Our patient appears to be the first proven pre-pubertal case of TS featuring no Y chromosome material but somatic *KIT* activation as the driver of oncogenesis. Previously, Gravholt et al. reported a single case of a metastatic embryonal carcinoma in a 40-year-old female with a 45,X chromosomal constitution without signs of the Y chromosome. Remarkably, whole exome sequencing revealed that the tumor harbored a gain-of-function pathogenic variant in exon 13 of the *KIT* gene (namely c.1965T>A; p.N655K; NM 000222.2) and additional putative somatic pathogenic variants in the *AKT1* and *ZNF358* genes, as well as a few genomic copy-number variations of uncertain importance [26]. The patient was successfully treated according to the poor prognosis germ cell tumor group protocol consisting of multimodal chemotherapy and eventual surgical debulking. For disease relapse, it seems that the use of imatinib or other tyrosine kinase inhibitors could provide a therapeutic option in tumors with KIT alternations [26,27,28].

Gonadal dysgenesis in TS cases may be due to the impairment of theca cell–oogonia interactions during gonadal development and differentiation during early fetal development, when female germ cells proceed into meiosis and begin folliculogenesis. In neonates, theca cell lineage requires both granulosa cells and oocytes through multicellular interactions via GDF9 and Hh signaling. In the absence of the Hh ligand, theca progenitor cells fail to differentiate into androgen-producing theca cells, and ovarian folliculogenesis is disrupted. The studied tumor showed the expression of Hh signaling pathway proteins with features of this pathway’s activation. The nuclear expression of GLI1 is also evidence of pathway activation [29]. No pathogenic variants in the key SHH pathway genes (*DHH*, *IHH*, *SHH*, *PTCH1*, *SMO*, *SUFU*, *GLI1*, *GLI2*, and *GLI3*) were detected in the molecular analysis. SHH expression was present within the cytoplasm of the neoplastic cells, which suggests an autocrine function in signaling [29]. The observed alteration of the SHH pathway is not exclusive for this case of dysgerminoma, as it was also previously reported in intracranial germinomas [30]. Interestingly, Hedgehog signaling has been found in the intestinal Cajal cells and is upregulated in gastrointestinal tumors (GISTs), causing GLI-mediated *KIT* expression, irrespective of *KIT*/*PDGFRA* mutational status [31].

In summary, even in the absence of chromosome Y-fragments, GTCs may develop in otherwise streaky gonads in TS patients through somatic KIT gene activation with probable supporting SHH pathway activation. Such molecular background constitutes a therapeutic target in disseminated high risk tumors.

## Figures and Tables

**Table 1:** Custom-designed NGS-based gene panel.

Hh Signaling Pathway Genes (Canonical)	Genes Associated with Hh Signaling Pathway	KIT/RAS Signaling Pathway Genes	mTOR Signaling Pathway Genes	Other Genes
DHH, IHH, SHH, PTCH1, SMO, SUFU, GLI1 GLI2 GLI3	DISP2, HHIP, LRP2, PTCH2, PTCHD1, ZIC1, ZIC2	KIT, KRAS, NRAS, HRAS, CBL	MTOR, PTEN	FGFR3, ERBB4
